# Impact of Different Surfactants on Oral Bioavailability of Paclitaxel/HPMC-AS Amorphous Solid Dispersion

**DOI:** 10.3390/pharmaceutics17111487

**Published:** 2025-11-18

**Authors:** Chenzhao Zhang, Siyi Mao, Jinhua Yuan, Xiuzhen Ma, Aiya Xing, Xiaoling Liu, Yuejie Chen

**Affiliations:** 1 School of Pharmacy, Minzu University of China, Beijing 100081, China; 23302008@muc.edu.cn (C.Z.); 24302083@muc.edu.cn (S.M.); 23302010@muc.edu.cn (J.Y.); 25302185@muc.edu.cn (X.M.); 22301902@muc.edu.cn (A.X.); 2 School of Life Sciences, Beijing University of Chinese Medicine, Beijing 102401, China; 3 Key Laboratory of Mass Spectrometry Imaging and Metabolomics (Minzu University of China), State Ethnic Affairs Commission, Beijing 100081, China

**Keywords:** paclitaxel (PTX), poorly water-soluble drug, oral bioavailability, amorphous solid dispersion (ASD), surfactant

## Abstract

**Objectives:** Surfactants are commonly incorporated into amorphous solid dispersions (ASDs) to improve manufacturing and enhance the dissolution of poorly water-soluble drugs. However, their impact on in vitro dissolution, in vivo bioavailability, and in vitro-in vivo correlation (IVIVC) remains poorly understood, impeding the rational design of ASDs. This study aimed to elucidate the impact of six surfactants: anionic sodium lauroyl glutamate (SLG), sodium taurocholate (NaTC), sodium lauryl sulfate (SLS), and non-ionic polysorbate 80 (TW80), poloxamer 188 (P188), and polyoxyethylene lauryl ether (Brij-35), on the performance of paclitaxel (PTX)/HPMC-AS ASD. **Methods:** Binary PTX/HPMC-AS and ternary PTX/HPMC-AS/surfactant ASDs were prepared via rotary evaporation for FT-IR study. For dissolution and pharmacokinetic studies, low drug-loading formulations were prepared by physically blending PTX/HPMC-AS ASD with surfactants. Drug–polymer–surfactant interactions were investigated using NMR and FT-IR techniques. Dissolution performance was systematically evaluated by analyzing: (1) solubility of crystalline PTX in HPMC-AS/surfactant solutions; (2) supersaturation sustaining capacity in HPMC-AS/surfactant solutions; (3) surfactant effects on ASD dissolution and supersaturation generation; and (4) phase transformation during ASD dissolution. In vivo bioavailability was assessed in rats. **Results:** Findings revealed surfactant-specific effects: (1) SLG and P188 minimally affected bioavailability of PTX/HPMC-AS ASD (*p* > 0.05), consistent with their negligible effect on dissolution, attributable to incompatibility with PTX/HPMC-AS and weak molecular interactions; (2) TW80 significantly reduced bioavailability (*p* < 0.001) by inducing crystallization; thereby diminishing the amorphous advantage; (3) NaTC, Brij-35, and SLS markedly increased bioavailability (*p* < 0.001), owing to their compatibility with PTX and HPMC-AS, which enhanced dissolution and maintained amorphous state of precipitates. Surfactants appear to modulate ASD performance by governing supersaturation generation in solution and maintaining amorphous stability in the undissolved solid. **Conclusions:** The dissolution and bioavailability of ASDs are fundamentally controlled by compatibility between drug, polymer, and surfactant. Surfactant selection critically impacts ASD bioavailability. Comprehensive dissolution characterization, including supersaturation kinetics and precipitate phase analysis, enables prediction of bioavailability. Integrating molecular-level interaction analysis with multidimensional dissolution profiling is therefore essential for rational ASD design.

## 1. Introduction

Oral administration remains the preferred route for drug delivery due to its convenience and high patient compliance. However, the low aqueous solubility of many drugs often impedes gastrointestinal absorption, leading to poor oral bioavailability and suboptimal clinical efficacy [1]. This is particularly concerning in modern drug discovery, where approximately 90% of new chemical entities exhibit poor solubility [2]. To address this limitation, supersaturating drug delivery systems, particularly amorphous solid dispersions (ASDs), have been developed to enhance the dissolution and oral absorption of insoluble drugs [3,4]. In ASDs, drug molecules are molecularly dispersed within a polymeric matrix, stabilizing the amorphous form, which possesses higher apparent solubility than its crystalline counterpart [5]. Surfactants are frequently incorporated into ASD formulations to improve wettability, enhance dissolution, and facilitate manufacturability [6,7,8]. Consequently, a thorough understanding of surfactant impact on both in vitro dissolution and in vivo bioavailability is vital for the rational design of ASD formulations.

However, the influence of surfactants on ASD dissolution and bioavailability is complex and hard to predict. Although in vitro studies consistently report enhanced dissolution performance upon surfactant addition [9,10,11], the underlying mechanisms are multifaceted. Surfactants lower surface tension and improve wettability, facilitating rapid ASD dissolution and generating drug supersaturation levels that exceed crystalline solubility, which is the prerequisite for enhanced absorption [12]. Crucially, the formation and stability of drug-rich nanodroplets via liquid–liquid phase separation (LLPS) are essential for maintaining supersaturation [13,14]. Surfactants can stabilize these nanodroplets and prolong supersaturation further through micellar encapsulation [15,16]. These stabilized nanodroplets act as reservoirs, replenishing free drug absorbed across the intestinal epithelium and thereby promoting trans-epithelial flux and intestinal uptake [17,18,19].

No clear correlation exists between surfactant physicochemical properties and their performance in ASDs [20]. This complexity arises from surfactants’ dualistic effects on supersaturation stability: while enhancing initial dissolution, the resulting high supersaturation inherently increases precipitation risk [21,22]. Surfactant efficacy is concentration-dependent, governed by the critical micelle concentration (CMC). Below CMC, surfactants can adsorb onto nascent nuclei, potentially accelerating crystallization; above CMC, micellar encapsulation may inhibit crystal growth but also reduce the free drug concentration available for absorption [23,24]. Crucially, robust water-resistant drug–polymer interactions are essential for stabilizing supersaturation by forming nano-species and inhibiting crystallization of amorphous drug precipitates during ASD dissolution [25,26]. Surfactants, however, can compete with the drug for polymer binding sites, potentially disrupting these stabilizing interactions and ultimately reducing oral absorption [27].

In vivo bioavailability studies of surfactant-containing ASDs reveal inconsistent results. For instance, Mane et al. demonstrated that a ternary ASD incorporating Eudragit L100 and sodium lauryl sulfate (SLS) significantly enhanced both solubility and oral absorption of docetaxel [28]. Conversely, Chen et al. reported that SLS addition competitively inhibited the crystallization-inhibiting function of HPMC-AS, significantly reducing the bioavailability of a posaconazole ASD [27]. These conflicting findings highlight that the mechanisms by which different surfactants influence ASD dissolution dynamics and ultimate bioavailability remain inadequately understood.

This study therefore aimed to elucidate the impact of different surfactants on the in vitro dissolution and in vivo absorption of paclitaxel/HPMC-AS ASD, as well as the correlation between them. A key objective was to mechanistically clarify how surfactants regulate ASD dissolution and oral bioavailability, thereby establishing a rational framework for surfactant selection in ASD formulation design and advancing in vitro-in vivo correlation (IVIVC) for bioavailability prediction. Paclitaxel (PTX), a broad-spectrum natural anticancer drug exhibiting poor intrinsic aqueous solubility (~0.3 μg/mL) and high lipophilicity (logP ~3.2) [29], was selected as a Biopharmaceutics Classification System (BCS) Class IV model drug. Hydroxypropyl methylcellulose acetate succinate (HPMC-AS) served as the polymeric carrier due to its established efficacy in enhancing solubility and inhibiting drug crystallization within ASDs [30]. To systematically evaluate the impact of surfactant properties, six commonly used surfactants with diverse physicochemical characteristics were selected: the anionic surfactants sodium lauroyl glutamate (SLG), sodium taurocholate (NaTC), and sodium lauryl sulfate (SLS), along with the non-ionic surfactants polysorbate 80 (TW80), poloxamer 188 (P188), and polyoxyethylene lauryl ether (Brij-35). This selection encompasses a broad spectrum of surfactant traits, including charge type, molecular structure, hydrophilic-lipophilic balance (HLB), and biological relevance. Specifically, NaTC is a natural bile acid surfactant used to simulate physiological conditions; SLS is a strong anionic surfactant with high solubilizing capacity, offering a contrast to the milder SLG; P188 is a non-ionic triblock copolymer that differs from the monoalkyl-structured TW80 and Brij-35 in both molecular architecture and steric stabilization properties. SLG is an anionic surfactant of cosmetic grade that exhibits unique and mild properties. These surfactants were selected to evaluate the applicability of the predictive framework developed for rational surfactant selection in ASD formulations.

Nuclear magnetic resonance (NMR) and Fourier transform infrared spectroscopy (FT-IR) were employed to characterize intermolecular interactions between the drug, polymer, and surfactants in both solution and solid states. Surfactant efficacy was further evaluated based on: (1) drug solubilization capacity, (2) supersaturation maintenance capability, and (3) dissolution behavior, including solution dissolution kinetics and precipitate phase transformations. Finally, in vivo pharmacokinetics studies assessed the impact of surfactants on ASD bioavailability.

## 2. Materials and Methods

### 2.1. Materials

**Figure 1:**
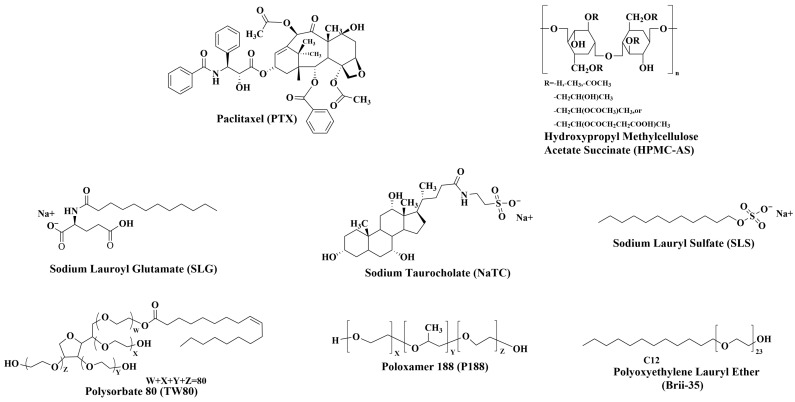
Chemical structures of the model drug (PTX), polymer carrier (HPMC-AS), and investigated surfactants (SLG, NaTC, SLS, TW80, P188, and Brij-35).

Paclitaxel (PTX), sodium lauroyl glutamate (SLG), sodium taurocholate (NaTC), sodium lauryl sulfate (SLS), polysorbate 80 (TW80), polyoxyethylene lauryl ether (Brij-35), and buffer salts for dissolution media preparation were purchased from Macklin Biochemical Co., Ltd. (Shanghai, China). Poloxamer 188 (Kolliphor^®^ P188) was obtained from BASF SE (Ludwigshafen, Germany). Hydroxypropyl methylcellulose acetate succinate (HPMC-AS, MF grade), selected as the carrier polymer due to its optimized performance in ASDs, was kindly provided by Shin-Etsu Chemical Co., Ltd. (Tokyo, Japan). Ethanol (analytical reagent grade, ≥99%), methanol (HPLC grade, ≥99.9%), and acetonitrile (LC-MS/MS grade, ≥99.9%) were obtained from Energy Chemical (Shanghai, China). The chemical structures of PTX, HPMC-AS, and the surfactants are presented in Figure 1.

### 2.2. Methods

#### 2.2.1. Preparation of PTX ASDs

PTX ASDs were prepared using distinct methods for specific applications.

(1) High drug-loading ASDs with surfactant incorporated during preparation (for FT-IR Studies): Binary PTX/HPMC-AS (1:1, *w*/*w*; 50 wt.% drug) and ternary PTX/HPMC-AS/surfactant ASDs (1:1:1, *w*/*w*/*w*; ~34 wt.% drug) were prepared by rotary evaporation to achieve molecular-level homogeneity. High drug loading promotes intimate drug–polymer–surfactant contact for intermolecular interactions while minimizing excipient interference in FT-IR spectra. Preparation: Stoichiometric mixtures of PTX, HPMC-AS, and surfactants (where applicable) were dissolved in ethanol, rotary-evaporated under reduced pressure, vacuum-dried (≥24 h), and stored at −20 °C prior to FT-IR analysis. For comparison, physical mixtures (PMs) of amorphous PTX, HPMC-AS, and surfactant (if used) in a 1:1:1 (*w*/*w*/*w*) ratio were also prepared. These components were sieved through a 0.3 mm sieve and blended until a homogeneous mixture was obtained. These samples were designated as PTX/HPMC-AS PM or PTX/HPMC-AS/surfactant PM and served as controls in the FT-IR study. The ASD and PM systems contain identical components in comparable quantities, but differ fundamentally in component intimacy and intermolecular interactions. In the PM system, components are physically mixed without intimate contact and do not form intermolecular interactions. In contrast, the ASD system achieves molecular-level mixing, enabling the formation of specific intermolecular interactions. FT-IR spectroscopy was employed to investigate solid-state drug–polymer–surfactant interactions, which serve as an indicator of compatibility among the components.

(2) Low drug-loading ASD formulations with surfactant added by physical blending (for Dissolution and Pharmacokinetic Studies): First, a PTX/HPMC-AS ASD (1:4, *w*/*w*; 20 wt.% drug) was produced. This ASD was then physically blended with various surfactants at 5:2 mass ratio (ASD: surfactant), yielding PTX/HPMC-AS/surfactant = 1:4:2 (*w*/*w*/*w*; ~14.3 wt.% drug). For clarity, the formulations are abbreviated as: ASD (PTX/HPMC-AS ASD); ASD + SLG (PTX/HPMC-AS ASD + SLG); ASD + NaTC (PTX/HPMC-AS ASD + NaTC); ASD + SLS (PTX/HPMC-AS ASD + SLS); ASD + TW80 (PTX/HPMC-AS ASD + TW80); ASD + P188 (PTX/HPMC-AS ASD + P188); ASD + Brij-35 (PTX/HPMC-AS ASD + Brij-35). The surfactant was introduced via physical blending, for it is a simpler and more industrially relevant process. The 1:4:2 (*w*/*w*/*w*) ratio was selected based on preliminary optimization studies, which indicated that this composition improves drug dissolution and supersaturation maintenance in aqueous media, making it suitable for in vitro dissolution and in vivo pharmacokinetic studies.

It is important to note that, while the formulations used for solid-state characterization and performance testing were prepared differently, they were strategically designed to probe complementary aspects of surfactant function, from fundamental molecular interactions governing formulation mechanism to overall biopharmaceutical performance in vitro and in vivo.

#### 2.2.2. Characterization of Intermolecular Interactions

##### Nuclear Magnetic Resonance (NMR)

^1^H NMR was performed to investigate solution-state interactions. Pure PTX, binary PTX/HPMC-AS (1:1, *w*/*w*), and ternary PTX/HPMC-AS/surfactant mixtures (SLG, NaTC, SLS, TW80, P188, or Brij-35; 1:1:1, *w*/*w*/*w*) were dissolved in deuterochloroform (CDCl_3_) at 5.0 mg/mL per component. Spectra were acquired using a Bruker AV-400 spectrometer (400 MHz; Bruker Corporation, Rheinstetten, Germany) with tetramethylsilane (TMS) as internal standard (δ = 0 ppm).

##### Fourier Transform Infrared Spectroscopy (FT-IR)

FT-IR characterized solid-state interactions. Pure amorphous PTX, HPMC-AS, PTX/HPMC-AS PM (1:1, *w*/*w*), PTX/HPMC-AS/surfactant PM (1:1:1, *w*/*w*/*w*), PTX/HPMC-AS ASD (1:1, *w*/*w*), and PTX/HPMC-AS/surfactant ASDs (1:1:1, *w*/*w*/*w*) were analyzed. Spectra were recorded on a Bruker Vertex 70 spectrometer (Bruker Corporation, Ettlingen, Germany) from 600 to 4000 cm^−1^ (128 scans, 4 cm^−1^ resolution).

#### 2.2.3. In Vitro Dissolution Studies

##### Equilibrium Solubility of Crystalline PTX

The equilibrium solubility of crystalline PTX was determined in PBS (pH 7.0) containing: single HPMC-AS (2, 4, or 8 mg/mL); single surfactant (SLG, NaTC, SLS, TW80, P188, or Brij-35; 1, 2, or 4 mg/mL); or HPMC-AS/surfactant combinations. Method: Excess crystalline PTX was suspended in each media, vortex-mixed (1 min), sonicated (10 min), and incubated at 37 °C for 24 h. After equilibration, supernatants were filtered (0.22 µm) and analyzed by HPLC-UV for drug quantification. All experiments were performed in triplicate.

##### Supersaturation Maintenance Assessment

Supersaturation maintenance capacity was evaluated in PBS containing: single HPMC-AS (4 mg/mL); single surfactants (2 mg/mL); or HPMC-AS/surfactant combinations (4 + 2 mg/mL). Method: 0.2 mL PTX stock solution (50 mg/mL in DMSO) was added to 10 mL PBS (final PTX concentration = 1 mg/mL). Solutions were maintained at 37 °C with agitation. Aliquots (0.5 mL) were withdrawn at 10 min, 0.5, 1, 2, and 4 h, immediately centrifuged (10,000 rpm, 3 min), and analyzed by HPLC-UV for drug quantification. All experiments were conducted in triplicate.

##### ASD Dissolution Under Non-Sink Conditions

The dissolution performance of (i) PTX/HPMC-AS ASD (1:4, *w*/*w*); (ii) PTX/HPMC-AS ASD + surfactant formulations were investigated under non-sink condition. Method: ASD formulations (50 mg ASD + 20 mg surfactant (if used)) were added to 10 mL PBS (final PTX concentration = 1 mg/mL) and agitated at 37 °C. At specified timepoints (10 min, 0.5, 1, 2, 4 h), aliquots (0.5 mL) were withdrawn and centrifuged (10,000 rpm, 3 min). Samples were processed as follows: Supernatant was analyzed by HPLC-UV for drug quantification; Precipitate (collected at 0.5, 2, 4 h) was vacuum-dried for crystallinity analysis by PXRD. All experiments were performed in triplicate.

##### Powder X-Ray Diffraction (PXRD)

PXRD analysis characterized crystallinity in freshly prepared ASDs and their dissolution precipitates, using pure crystalline PTX and individual surfactants (SLG, NaTC, SLS, Tween 80, P188, and Brij-35) as controls. Samples were mounted on silicon zero-background holders and analyzed using a Rigaku SmartLab diffractometer (Tokyo, Japan) with Cu K_α_ radiation (40 kV, 40 mA). Scans were performed from 5° to 45° (2θ) at 10°/min under ambient conditions.

#### 2.2.4. In Vivo Pharmacokinetic Study

##### Animals

Male Sprague–Dawley rats (200 ± 20 g; SPF Biotechnology Co., Ltd., Beijing, China) were acclimatized under controlled conditions (temperature: 22 ± 1 °C; humidity: 55 ± 5%; 12-h light/dark cycle) with free access to food and water. All experimental protocols were approved by the Institutional Animal Care and Use Committee of the Air Force Medical Center, China.

##### Pharmacokinetic Protocol

PTX/HPMC-AS ASD (1:4, *w*/*w*) or PTX/HPMC-AS ASD + surfactant formulations were orally administered to SD rats to investigate their bioavailability performance. Method: Forty rats were fasted overnight (12 h) with free access to water and randomly divided into eight groups (*n* = 5/group): (i) Crystalline PTX suspension; (ii) surfactant-free PTX/HPMC-AS ASD; (iii) ASD + SLG; (iv) ASD + NaTC; (v) ASD + SLS; (vi) ASD + TW80; (vii) ASD + P188; (viii) ASD + Brij-35. Formulations were suspended in PBS and administered orally at 40 mg/kg PTX-equivalent dose. Serial blood samples (~200 μL) were collected via the retro-orbital venous plexus at 0.17, 0.25, 0.5, 0.75, 1, 1.5, 2, 4, and 24 h post-dosing. Samples were immediately centrifuged (2000 rpm, 5 min, 4 °C), and plasma was stored at −80 °C until LC-MS/MS analysis for drug quantification.

#### 2.2.5. Quantitative Determination Methods

##### HPLC-UV Analysis

PTX quantification in in vitro dissolution studies employed a Waters 2695 Alliance HPLC system (Milford, MA, USA) with Waters 2998 PDA detector. Samples were diluted 1:2 (*v*/*v*) with methanol prior to analysis. Chromatographic separation was performed using an Acquity UPLC BEH C_18_ column (2.1 × 50 mm, 1.7 μm; Waters) maintained at 25 °C. The isocratic mobile phase (0.1% formic acid in water–methanol = 80:20, *v*/*v*) flowed at 1.0 mL/min. Detection wavelength: 227 nm; injection volume: 10 μL; total run time: 6 min (PTX retention time: 3.92 min). Calibration curves (5.0–500.0 μg/mL) exhibited excellent linearity (R^2^ > 0.999). Data processing used Empower^®^3 software (Waters).

##### LC-MS/MS Method

PTX plasma quantification utilized a SCIEX QTRAP^®^ 5500 LC-MS/MS system with ExionLC™ AC (Framingham, MA, USA). Sample preparation: 50 μL of plasma was extracted with 650 μL ethyl acetate containing internal standard (IS) acetaminophen (35 ng/mL), vortex-mixed (3 min), and centrifuged (10,000 rpm, 10 min, 4 °C). The organic layer was transferred and evaporated under nitrogen. Reconstituted samples were analyzed using a Waters Acquity UPLC HSS T3 column (100 × 2.1 mm, 1.8 μm) at 40 °C. Gradient elution (mobile phase A: 0.1% formic acid in water; B: acetonitrile) at 0.3 mL/min: 0–4.0 min: 10% B; 4.0–9.5 min: 10 → 95% B; 9.5–12.0 min: 95 → 10% B. Positive ESI-MS/MS detection with MRM transitions: *m*/*z* 876.5 → 308.1 (DP: 95.0 V; CE: 42.0 V) for PTX; *m*/*z* 152.0 → 110.0 (DP: 80.0 V; CE: 15.0 V) for acetaminophen (IS). Retention times: PTX = 4.85 min; IS = 2.65 min. Calibration range: 1.0–500.0 ng/mL (R^2^ > 0.999).

#### 2.2.6. Data Analysis

Pharmacokinetic parameters were calculated via non-compartmental analysis (DAS 2.0; Boying Corp., Beijing, China). Data presented as mean ± SD. Statistical significance (*p* < 0.05) determined by Student’s *t*-test using SPSS 13.0 (Chicago, IL, USA).

## 3. Results

### 3.1. Interactions Between Drug, Polymer and Surfactant

Non-covalent drug–polymer interactions critically govern ASD physical stability, dissolution behavior, and bioavailability [31,32]. Strong intermolecular associations inhibit amorphous phase separation and recrystallization during manufacturing/storage while enhancing dissolution-generated supersaturation to improve gastrointestinal absorption [33,34]. We therefore characterized drug–polymer interactions and surfactant-mediated alterations using complementary spectroscopic techniques. ^1^H NMR and FT-IR spectroscopy were employed to probe intermolecular interactions in solution and solid states, respectively. Solution NMR elucidates interaction mechanisms under molecularly dissolved conditions, mimicking post-administration dissolution dynamics. Solid-state FT-IR predicts ASD storage stability and dissolution performance.

#### 3.1.1. NMR Analysis of Intermolecular Interactions

**Figure 2:**
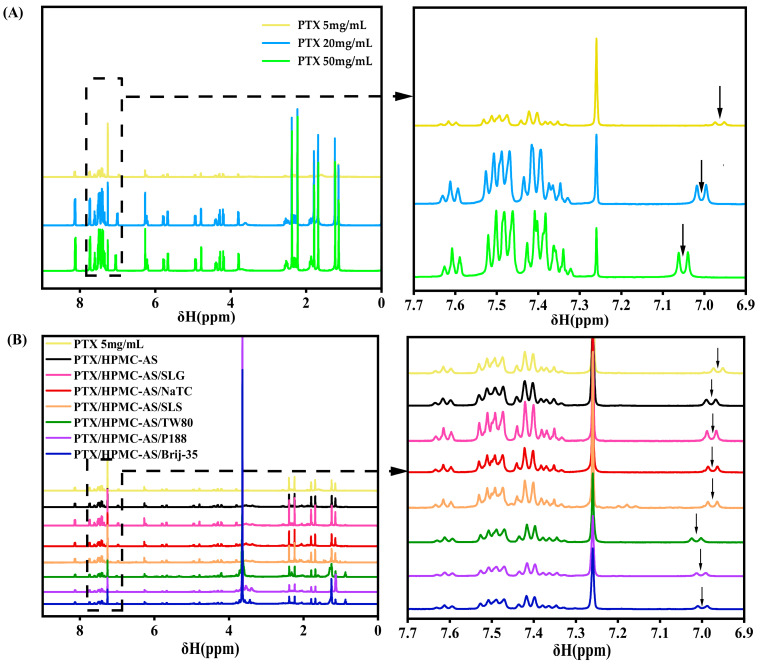
^1^H NMR spectra (in CDCl_3_) of: (**A**) pure PTX at concentrations of 5, 20, and 50 mg/mL; (**B**) binary PTX/HPMC-AS (1:1, *w*/*w*) and ternary PTX/HPMC-AS/surfactant (1:1:1, *w*/*w*/*w*) systems, with each component at 5 mg/mL. The region (δ 6.90–7.70 ppm) is expanded to facilitate comparison of the chemical shift changes in the PTX -NH proton. Black arrows indicate the downfield shift associated with increasing PTX concentration (**A**), or the shift induced by the addition of HPMC-AS and/or surfactants (**B**).

^1^H NMR provides atomic-resolution insights into site-specific molecular interactions through changes in chemical shift. To investigate PTX-HPMC-AS binding and surfactant effects, we analyzed the following samples: pure PTX at 5, 20, 50 mg/mL, binary PTX/HPMC-AS (1:1, *w*/*w*), and ternary PTX/HPMC-AS/surfactant (SLG, NaTC, SLS, TW80, P188, or Brij-35; 1:1:1, *w*/*w*/*w*) systems, with each component dissolved in CDCl_3_ at 5 mg/mL. The corresponding ^1^H NMR spectra are presented in Figure 2. CDCl_3_ was selected as the solvent for its dissolution capacity and minimal hydrogen-bond interference, preserving detectable interactions.

Figure 2A displays ^1^H NMR spectra of pure PTX at different concentrations. The -NH proton signal of pure PTX at 5 mg/mL (yellow line) appeared at δ 6.96 ppm (d, *J* = 8.8 Hz, ^1^H). This signal downfield shifted to δ 7.01 ppm at 20 mg/mL (blue line) and further to δ 7.05 ppm at 50 mg/mL (green line), while all other proton signals remained unchanged. This specific deshielding is attributed to enhanced intermolecular hydrogen bonding at higher concentrations, which reduces the electron density around the amide proton.

Figure 2B compares the ^1^H NMR spectra of drug/polymer/surfactant combinations. Upon the addition of HPMC-AS to PTX, the -NH proton signal (originally at δ 6.96 ppm) shifted slightly downfield to δ 6.98 ppm (black line), while other peaks remained unchanged, indicating hydrogen bond formation between PTX and HPMC-AS. In ternary PTX/HPMC-AS/surfactant systems, different surfactants induced distinct -NH signal shifts. The addition of anionic surfactants (SLG, NaTC, or SLS) did not alter the chemical shift observed in the binary PTX/HPMC-AS system (maintained at δ 6.98 ppm), confirming that these surfactants do not interfere with the hydrogen bonding between PTX and HPMC-AS in solution. In contrast, the incorporation of non-ionic surfactants (TW80, P188, or Brij-35) led to further downfield shifts to δ 7.01 ppm with TW80, and to δ 7.00 ppm with P188 and Brij-35. These observations imply a strengthening of hydrogen bonding in the presence of non-ionic surfactants.

#### 3.1.2. FT-IR Analysis of Intermolecular Interactions

**Figure 3:**
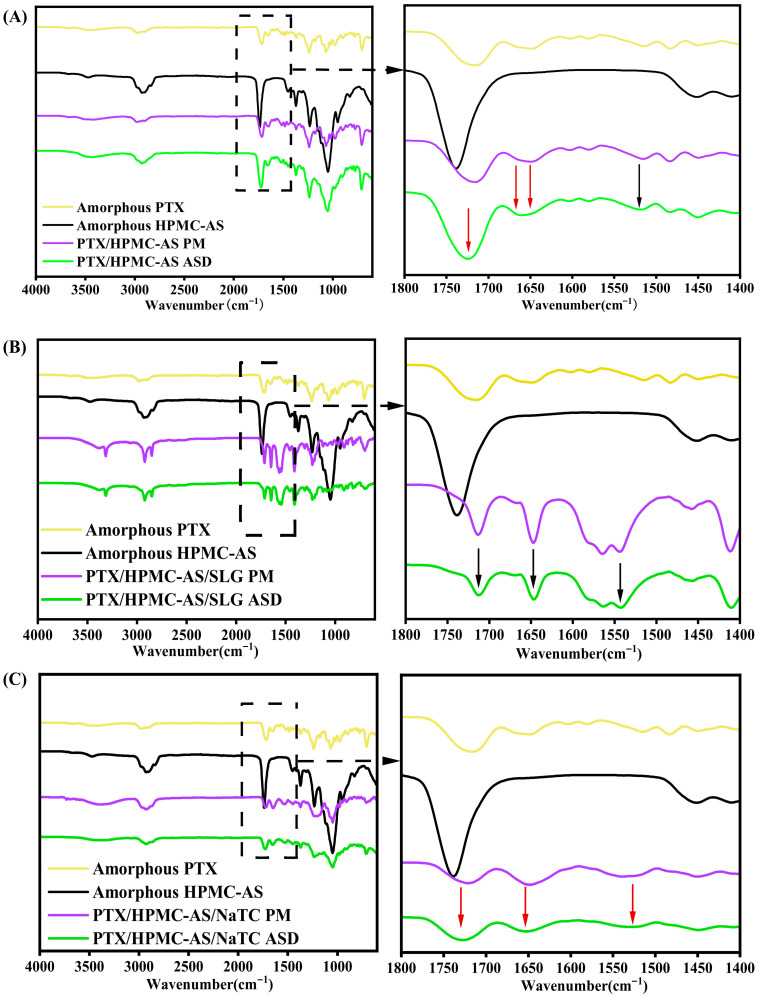

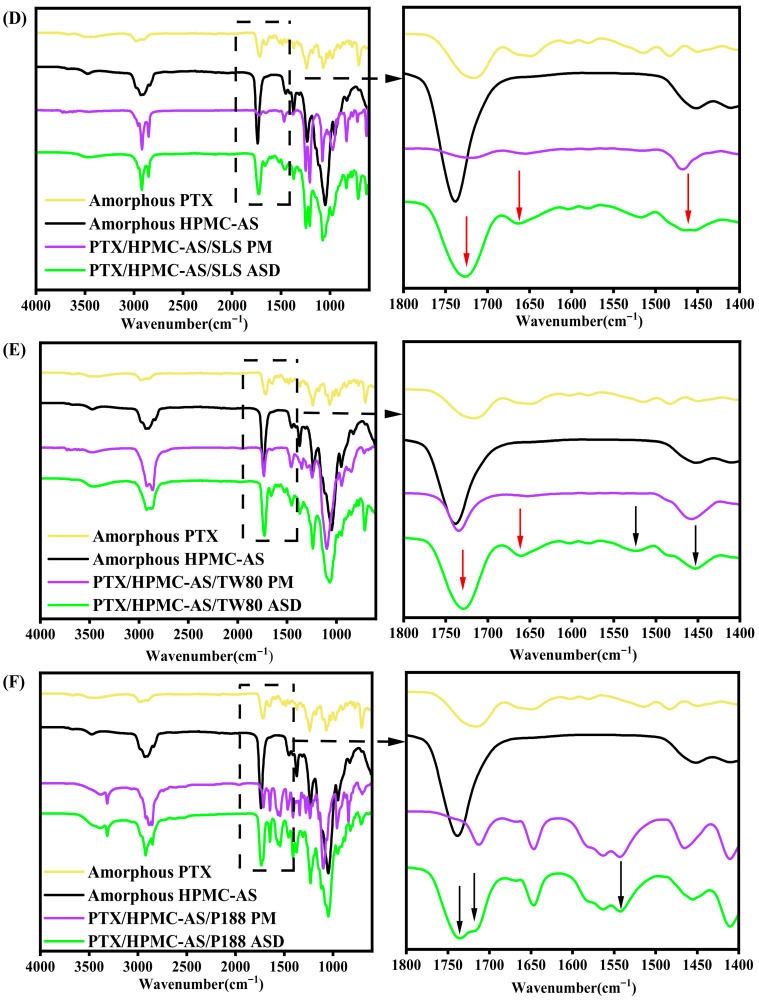

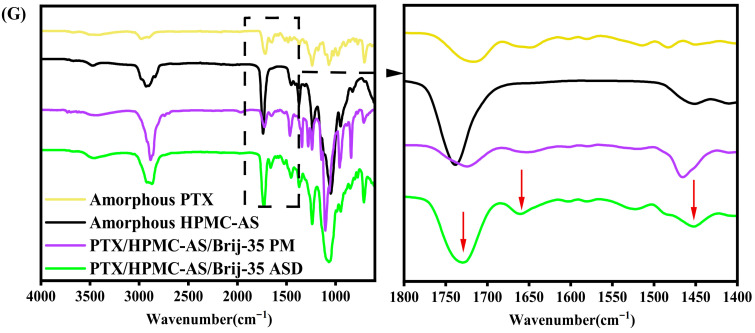
FT-IR spectra of ASDs and their corresponding controls (pure components or physical mixtures, PMs): (**A**) binary PTX/HPMC-AS systems; (**B**–**G**) Ternary PTX/HPMC-AS/surfactant systems with SLG, NaTC, SLS, TW80, P188, and Brij-35, respectively. The expanded region (1800–1400 cm^−1^) highlights the carbonyl and aromatic vibrational bands. Black arrows mark peaks consistent with pure components or PMs; red arrows indicate changes specific to ASDs.

FT-IR spectroscopy provides atomic-level characterization of solid dispersions by detecting absorption band alterations (e.g., shifts, intensity variations, emergence/disappearance) when functional groups participate in intermolecular interactions that alter vibrational frequencies [35]. This technique was applied to study the solid-state interactions among PTX, HPMC-AS, and surfactants in homogeneous binary and ternary ASDs. FT-IR analysis provides insights into the compatibility between components, which is closely correlated with the dissolution performance of ASDs. We investigated drug–polymer–surfactant interactions through comparative analysis of the pure amorphous components, binary PTX/HPMC-AS ASD, and ternary PTX/HPMC-AS/surfactant ASDs, alongside their corresponding physical mixtures (PM) as controls. The FT-IR spectra are presented in Figure 3. We need to note that, in these spectra, peaks unchanged between ASDs and their corresponding pure components or physical mixtures are marked with black arrows, while altered peaks are indicated with red arrows.

Figure 3A compares the spectra of amorphous PTX, HPMC-AS, PTX/HPMC-AS PM, and PTX/HPMC-AS ASD. The expanded region (1800–1400 cm^−1^) highlights carbonyl and aromatic vibrations. Amorphous PTX (yellow line) exhibited four characteristic carbonyl stretches: 1726 cm^−1^ (free C=O), 1714 cm^−1^ (H-bonded C=O), 1664 cm^−1^ (free benzoyl), 1649 cm^−1^ (H-bonded benzoyl). The higher intensity of the H-bonded peaks (1714 > 1726 cm^−1^; 1649 > 1664 cm^−1^) indicates significant dominance of intramolecular hydrogen bonding. Aromatic vibrations were observed at 1602, 1581, 1514, 1483, and 1452 cm^−1^; these may participate in strong π-π stacking interactions, as reported in the literature [36]. HPMC-AS (black line) showed an ester C=O stretch at 1738 cm^−1^ and CH_2_/CH_3_ bending at 1452 cm^−1^. The spectrum of the PTX/HPMC-AS PM (purple line) closely resembled that of the physical combination of pure amorphous PTX and HPMC-AS, indicating no significant intermolecular interactions between the components in PM system. In contrast, PTX/HPMC-AS ASD (green line) spectrum exhibited distinct features relative to both amorphous PTX and HPMC-AS. Key changes relative to PTX include the disappearance of the 1714 cm^−1^ peak, reduced intensity at 1649 cm^−1^, and enhanced intensities at 1726 and 1664 cm^−1^. Furthermore, the HPMC-AS carbonyl peak red-shifted to 1726 cm^−1^, overlapping the PTX peak. These spectral changes indicate that drug–polymer hydrogen bonding replaces drug–drug interactions. In contrast, the unchanged aromatic peaks suggest preservation of π-π stacking.

Figure 3B compares the spectra of PTX/HPMC-AS/SLG systems. The carbonyl and aromatic vibrations were similar between drug/polymer/SLG PM (purple line) and the corresponding ASD (green line), indicating no specific interactions formed between PTX, HPMC-AS, and SLG in ASD. Similarly, Figure 3F compares the spectra of PTX/HPMC-AS/P188 systems. The PTX/HPMC-AS/P188 ASD (green line) displayed two carbonyl peaks at 1738 cm^−1^ (identical to HPMC-AS) and 1714 cm^−1^ (identical to free PTX). Moreover, the aromatic region showed similar peaks to those of physical mixture (purple line). This additive nature of the spectrum suggests a lack of specific interactions between PTX, HPMC-AS, and P188 in the ASD.

In contrast, Figure 3E compares the PTX/HPMC-AS/TW80 system. ASD (green line) spectra displayed two peaks at 1730 cm^−1^ and 1662 cm^−1^, which differ from those of the pure components or their physical mixture (yellow, black, and purple lines), likely due to hydrogen bonding formation in the ASD. However, the aromatic region remained similar to pure amorphous PTX, indicating that HPMC-AS and TW80 do not interfere with the π-π stacking between drug molecules.

For the PTX/HPMC-AS/NaTC ASD (Figure 3C), PTX/HPMC-AS/SLS ASD (Figure 3D), and PTX/HPMC-AS/Brij-35 ASD (Figure 3G), new peaks emerged in both the carbonyl and aromatic regions, distinct from those of the pure components or physical mixtures. This indicates specific interactions formed among the drug, polymer, and surfactant in these three ASDs. Moreover, the presence of HPMC-AS and surfactants in these ASDs appears to disrupt the π-π stacking interactions between drug molecules.

In summary, NMR and FT-IR analyses consistently revealed the formation of intermolecular hydrogen bonding in both solution and solid states for the PTX/HPMC-AS and PTX/HPMC-AS/TW80 systems, indicating good molecular compatibility that facilitates intimate contact within the ASD matrix. Notably, neither of these systems interfered with π-π stacking between drug molecules. Conversely, no specific interactions were detected in the PTX/HPMC-AS/SLG and PTX/HPMC-AS/P188 ASDs, likely due to poor miscibility preventing molecular proximity during processing. In contrast, the PTX/HPMC-AS/NaTC, PTX/HPMC-AS/SLS, and PTX/HPMC-AS/Brij-35 ASDs exhibited enhanced hydrogen bonding networks, indicating superior ternary miscibility; moreover, drug’s π-π stacking interactions were also interfered with in these systems.

### 3.2. In Vitro Dissolution Performance of PTX Formulations

#### 3.2.1. Solubility of Crystalline PTX in PBS with Excipients

The solubilization capacity of HPMC-AS, surfactants, and their combinations was quantified (Table 1). Crystalline PTX exhibited negligible solubility in PBS (<0.1 μg/mL), while excipient systems showed differential enhancement. HPMC-AS alone (2–8 mg/mL) exhibited concentration-dependent improvement (6.6–10.7 μg/mL). Among the surfactants tested at 1–4 mg/mL, SLG and P188 offered minimal enhancement (SLG: 1.1–1.3 μg/mL; P188: 1.1–2.4 μg/mL). In contrast, others demonstrated greater efficacy: NaTC (3.6–10.1 μg/mL), SLS (3.5–17.0 μg/mL), Tween 80 (2.5–9.0 μg/mL), and Brij-35 (1.7–6.7 μg/mL).

HPMC-AS/surfactant combination systems revealed distinct behaviors. HPMC-AS with SLG, NaTC, or P188 produced merely additive effects. For example, the combination of HPMC-AS (8 mg/mL) and SLG (4 mg/mL) yielded a solubility of 12.0 μg/mL, comparable to the sum of the individual contributions (10.7 + 1.3 μg/mL). In contrast, strongly synergistic effects were observed for HPMC-AS combined with SLS, Tween 80, or Brij-35. At concentrations of 8 mg/mL HPMC-AS and 4 mg/mL surfactant, these combinations achieved PTX solubilities of 177.3 μg/mL (SLS), 132.4 μg/mL (Tween 80), and 128.3 μg/mL (Brij-35).

PTX’s aqueous insolubility originates from self-assembly via hydrogen bonding and π-π stacking. HPMC-AS disrupts aggregation through competitive hydrogen bonding. The lack of synergy within HPMC-AS/SLG and HPMC-AS/P188 combinations aligns with the poor ternary miscibility observed in the NMR and FT-IR studies, which likely prevents effective interaction with PTX. The HPMC-AS/NaTC combination also lacked synergy in solubilizing crystalline PTX, which may be attributed to the unique structure of NaTC as a bile salt favoring the formation of its own micellar or colloidal structures, thereby solubilizing PTX independently rather than cooperatively with the polymer. In contrast, the pronounced synergy within HPMC-AS/Tween 80, HPMC-AS/SLS, and HPMC-AS/Brij-35 systems can be attributed to their better compatibility, which successfully disrupts PTX self-association by interfering with both drug–drug hydrogen bonding and π-π stacking, as evidenced by the spectral changes.

#### 3.2.2. Effect of Excipients on PTX Supersaturation Maintenance

**Figure 4:**
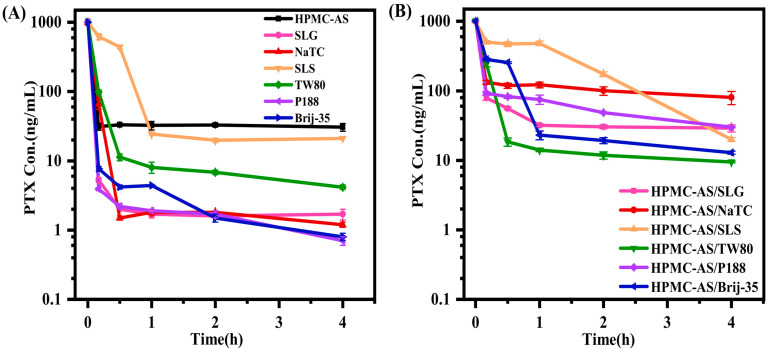
Supersaturation maintenance profiles of PTX in PBS with: (**A**) Individual components (4 mg/mL HPMC-AS or 2 mg/mL surfactants; (**B**) HPMC-AS/surfactant combinations (4 + 2 mg/mL). (mean ± SD, *n* = 3).

Dissolution of ASDs generates supersaturation that enhances oral absorption of poorly water-soluble drugs when stabilized [12]. We evaluated the supersaturation maintenance capacity of HPMC-AS (4 mg/mL), surfactants (2 mg/mL), and HPMC-AS/surfactant combinations (4 + 2 mg/mL), using an initial PTX concentration of 1 mg/mL (Figure 4).

PTX in PBS precipitated rapidly, falling below 0.1 μg/mL within 10 min. Figure 4A shows PTX supersaturation kinetics in individual components. HPMC-AS alone stabilized PTX at ~30 μg/mL (0.5–4 h) after an initial drop (black line). Among the surfactants, only SLS (orange line) effectively maintained a high supersaturation level for 0.5 h (437.9 μg/mL). The other surfactants (SLG, NaTC, Tween 80, P188, and Brij-35) failed to sustain drug supersaturation, with concentrations falling below 10 μg/mL within 0.5 h and continuing to decrease thereafter (pink, red, green, purple, and blue lines).

Figure 4B reveals the combination effects. The systems containing HPMC-AS/SLG and HPMC-AS/P188 combinations showed minimal ability to maintain supersaturation: over 90% of PTX precipitated rapidly, resulting in concentrations below 100 μg/mL within 10 min, followed by a further decrease to ~30 μg/mL at 4 h (pink and purple lines). The HPMC-AS/Tween 80 combination (green line) maintained high supersaturation for only 10 min (244.1 μg/mL), after which it decreased sharply to ~18 μg/mL by 0.5 h and continued to decline gradually to ~10 μg/mL at 4 h. In contrast, the HPMC-AS/NaTC system (red line) sustained drug concentrations between ~80–140 μg/mL throughout the experiment (10 min to 4 h). The HPMC-AS/SLS combination (orange line) effectively maintained supersaturation above 174.4 μg/mL for up to 2 h, before declining to 20 μg/mL by 4 h. The HPMC-AS/Brij-35 system (blue line) maintained high supersaturation for 0.5 h (255.0 μg/mL), after which it gradually decreased to ~13 μg/mL at 4 h.

Overall, the HPMC-AS/SLS, HPMC-AS/NaTC, and HPMC-AS/Brij-35 systems demonstrated superior supersaturation performance. This outstanding behavior can be attributed to the distinct mechanisms associated with each surfactant. In the HPMC-AS/SLS and HPMC-AS/Brij-35 systems, the strong synergy and good compatibility effectively disrupt PTX self-association, thereby inhibiting drug precipitation and maintaining a high degree of supersaturation over an extended period. In the case of HPMC-AS/NaTC, the unique structure of the bile salt enables efficient drug solubilization and gives rise to a slow yet sustained release profile. Together, these characteristics contribute to effective precipitation inhibition and prolonged supersaturation maintenance across all three systems.

#### 3.2.3. Dissolution Performance of PTX Formulations Under Non-Sink Conditions

Generating high supersaturation during ASD dissolution is essential for enhancing gastrointestinal drug absorption. We evaluated surfactants’ effects on PTX/HPMC-AS ASD dissolution under non-sink conditions (with ASDs loaded to achieve an initial PTX concentration of 1 mg/mL; ~10,000-fold supersaturation relative to its crystalline solubility <0.1 μg/mL).

**Figure 5:**
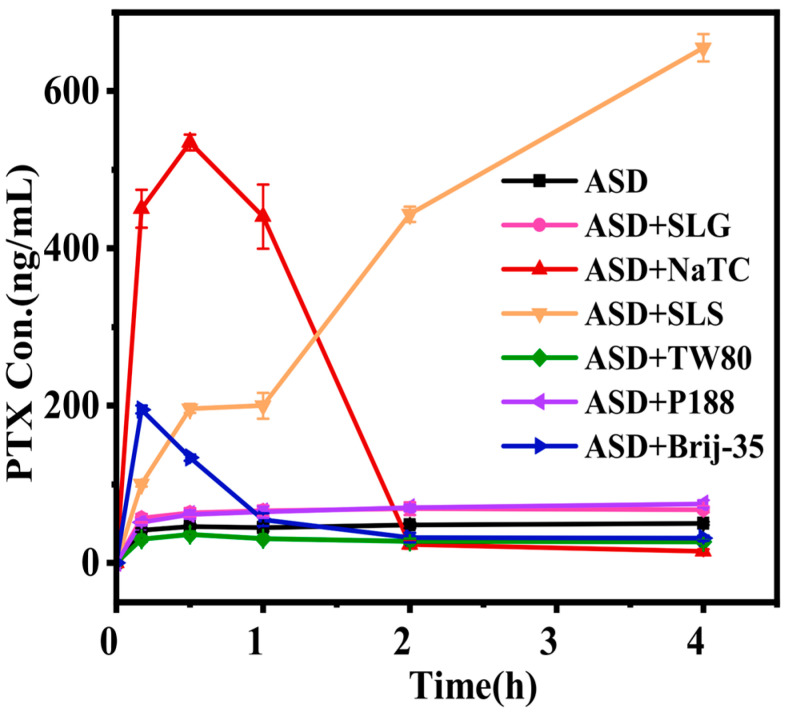
In vitro dissolution profiles of PTX formulations under non-sink conditions (initial PTX concentration = 1 mg/mL) (mean ± SD, *n* = 3).

Figure 5 compares the dissolution kinetics. The surfactant-free PTX/HPMC-AS ASD (black line) exhibited slow and incomplete dissolution, reaching only 41.8 μg/mL at 10 min and gradually increasing to 50.5 μg/mL over 4 h. The incorporation of surfactants distinctly influenced ASD dissolution. The addition of SLG (ASD + SLG, pink line) or P188(ASD + P188, purple line) led to only marginal improvements, with final concentrations of 68.0 μg/mL and 75.0 μg/mL, respectively, after 4 h. Notably, Tween 80 addition (ASD + TW80, green line) displayed the slowest dissolution rate, with only 30.2 μg/mL dissolved within 0.5 h, followed by crystallization and precipitation, yielding a final concentration of 26.7 μg/mL at 4 h.

In contrast, the incorporation of NaTC (ASD + NaTC, red line), SLS (ASD + SLS, orange line), or Brij-35 (ASD + Brij-35, blue line) markedly enhanced dissolution. ASD + NaTC dissolved rapidly upon aqueous contact, achieving 450.5 μg/mL within 10 min and maintaining this high level for up to 1 h, before declining sharply to 14.7 μg/mL by 4 h. Similarly, ASD + Brij-35 showed rapid initial release, reaching 195.2 μg/mL at 10 min and sustaining 134.0 μg/mL for 0.5 h, after which the concentration decreased to 31.6 μg/mL at 4 h. In comparison, ASD + SLS exhibited a different behavior: it dissolved more gradually in the first 10 min (100.7 μg/mL), but continued to release steadily over time, attaining a high concentration of 655.2 μg/mL by 4 h.

**Figure 6:**
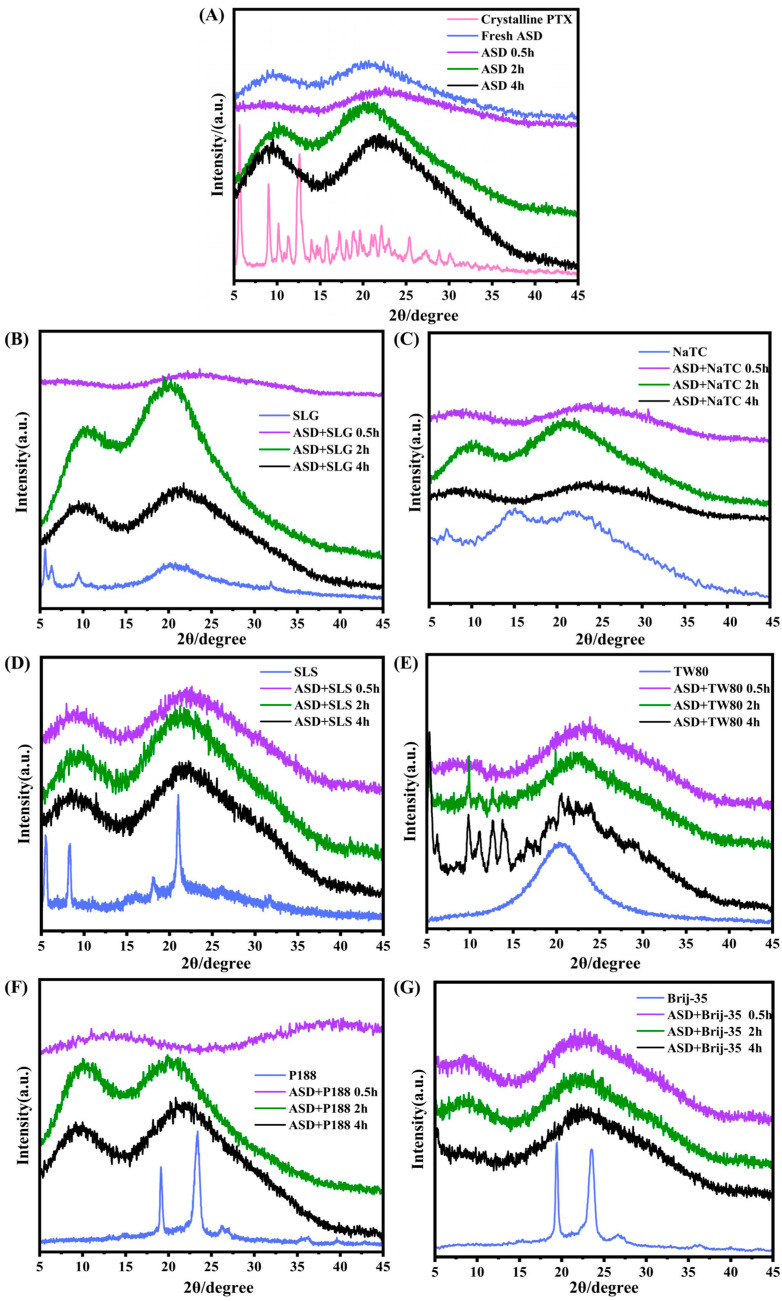
PXRD patterns of precipitates collected at time 0.5 h, 2 h and 4 h during dissolution of PTX formulations: (**A**) surfactant-free PTX/HPMC-AS ASD, (**B**–**G**) ASD + surfactant systems with SLG, NaTC, SLS, TW80, P188, and Brij-35, respectively. Crystalline PTX, individual surfactant, and freshly prepared ASD are included for comparison.

Notably, a significant portion of the dissolved drug precipitated during the dissolution process. Given that amorphous precipitates serve as high-energy reservoirs that enhance absorption [27,37], we analyzed precipitate crystallinity by PXRD. Precipitates were collected at 0.5, 2, and 4 h during ASD dissolution (Figure 6).

Figure 6A compares the precipitates from PTX/HPMC-AS ASD with crystalline PTX as a control. Crystalline PTX (pink line) exhibited characteristic sharp diffraction peaks, confirming its crystalline structure. The freshly prepared ASD (blue line) showed featureless pattern, confirming its amorphous nature. Precipitates from the surfactant-free ASD maintained amorphous characteristics throughout dissolution (purple, green, and black lines). Precipitates from ASD + SLG, ASD + NaTC, ASD + SLS, ASD + P188, and ASD + Brij-35 systems (Figure 6B–G) all exhibited featureless patterns at each time point, indicating that the amorphous state was preserved during the entire dissolution process. However, crystalline peaks emerged within 2 h for the ASD + Tween 80 formulation, indicating drug crystallization in precipitates (Figure 6E).

HPMC-AS inhibits crystallization through specific interactions with PTX, adsorbing onto drug surfaces to form interfacial barriers that retard crystal growth [38]. The surfactants differentially modulated this inhibition. SLG and P188 caused minimal disruption to amorphous stability, consistent with their negligible impact on PTX-HPMC-AS interactions and the limited improvement observed in the dissolution of corresponding ASD formulations.

In contrast, the addition of NaTC, SLS, or Brij-35 into the ASD significantly enhanced dissolution, generating a high degree of drug supersaturation. Although such supersaturated states are thermodynamically unstable and prone to precipitation and crystallization, these surfactants exhibited good compatibility with both PTX and HPMC-AS; the intermolecular interactions within these ternary systems likely contributed to maintaining the amorphous character of the precipitates. On the other hand, while Tween 80 also promoted initial dissolution and generated high supersaturation, it failed to effectively inhibit crystallization of the precipitated drug, leading to rapid conversion to the more stable crystalline form.

In summary, the dissolution studies demonstrate that the ASD + NaTC, ASD + SLS, and ASD + Brij-35 formulations delivered optimal performance, achieving high drug supersaturation, sustaining it for a sufficient duration, and maintaining the amorphous nature of the precipitates throughout the 4-h test.

### 3.3. Pharmacokinetics of PTX Formulations

**Figure 7:**
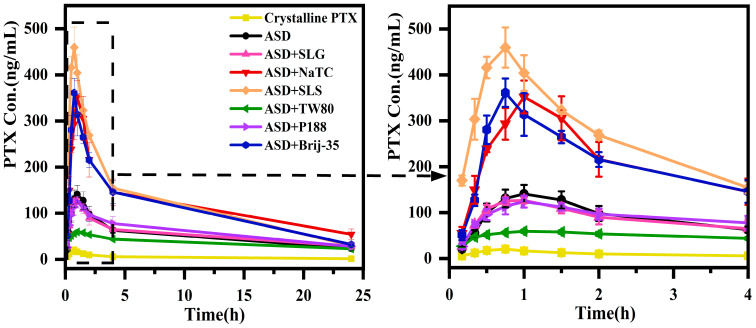
Mean plasma concentration vs. time profiles after oral administration of different formulations (40 mg/kg eq.) in rats (mean ± SD, *n* = 5).

The in vivo pharmacokinetics of crystalline PTX and ASD formulations were carried out in fasted male rats (40 mg/kg PTX eq.). Figure 7 shows plasma concentration-time profiles, and the corresponding pharmacokinetic parameters from non-compartmental analysis are summarized in Table 2. Note: Statistical significance was determined using Student’s *t*-test. The symbol # indicates comparison between crystalline PTX and PTX/HPMC-AS ASD; * indicates comparison between the surfactant-free ASD and ASD + surfactant formulations.

Overall, all ASD formulations significantly outperformed crystalline PTX in maximum plasma concentration (C_max_) and area under the curve (AUC_0–24hrs_) values. The surfactant-free PTX/HPMC-AS ASD exhibited a C_max_ of 140.6 ng/mL and an AUC_0–24hrs_ of 1231.2 ng·h/mL, representing 6.8-fold and 10.3-fold increases over crystalline PTX (C_max_ of 20.6 ng/mL; AUC_0–24hrs_ of 118.5 ng·h/mL), respectively. This significant enhancement in oral bioavailability is attributed to the improved dissolution performance of the ASD, specifically its ability to generate and maintain supersaturation, as well as with amorphous precipitates acting as dissolution reservoirs.

Different surfactants incorporation in ASD has different effects on its bioavailability performance. The addition of SLG (ASD + SLG) or P188 (ASD + P188) did not significantly alter bioavailability, as evidenced by C_max_ (128.3 and 124.0 ng/mL, respectively) and AUC_0–24hrs_ (1281.3 and 1420.4 ng·h/mL, respectively) values comparable to the surfactant-free ASD (*p* > 0.05). This finding is consistent with the in vitro dissolution results, where neither surfactant markedly affected dissolution kinetics, supersaturation generation, or precipitate transformation. In contrast, Tween 80 addition (ASD + TW80) significantly reduced bioavailability of ASD, decreasing C_max_ by 58% (140.6 to 59.7 ng/mL) and AUC_0–24hrs_ by 30% (1231.2 to 865.8 ng·h/mL) relative to the surfactant-free ASD (*p* < 0.001). This bioavailability reduction likely results from decreased drug supersaturation and precipitate crystallization during dissolution, and thus the loss of amorphous advantage. Conversely, the addition of NaTC (ASD + NaTC), SLS (ASD + SLS), or Brij-35 (ASD + Brij-35) significantly enhanced absorption. The ASDs achieved C_max_ values of 351.9, 459.6 and 361.1 ng/mL (increases of 2.5-, 3.3-, and 2.6-fold, respectively) and AUC_0–24hrs_ values of 2849.0, 2899.8, and 2612.6 ng·h/mL (increases of 2.3-, 2.4-, and 2.1-fold, respectively) compared to the surfactant-free ASD (*p* < 0.001).

The optimal oral absorption performance of ASD + NaTC, ASD + SLS, and ASD + Brij-35 correlated well with their in vitro dissolution profiles. The enhanced bioavailability originates from the dual functionality of these surfactants, simultaneously promoting supersaturation generation and prolonging its maintenance, while also preserving the amorphous state of the precipitates. This synergy ensures that drug molecules remain in a high-energy state that facilitates absorption.

## 4. Discussion

The impact of surfactants on both dissolution performance and ultimate bioavailability of ASDs represents a critical yet incompletely understood research domain. While previous studies have characterized surfactant effects on ASD dissolution and supersaturation [10,16,20], paradoxical negative impacts persist despite typically enhanced dissolution rates. Similarly, in vivo studies report conflicting evidence regarding surfactant-mediated bioavailability enhancement [19,27,28]. Critically, correlations between in vitro dissolution and in vivo bioavailability for surfactant-modified ASDs remain elusive, a fundamental gap impeding rational formulation design. This predictability challenge likely stems from surfactants’ complex interference with interdependent physicochemical processes governing ASD dissolution. Our study comprehensively investigates: (1) Mechanistic impacts of structurally diverse surfactants on ASD dissolution progression. (2) Molecular interactions driving observed behaviors. (3) In vitro–in vivo correlations for surfactant-containing ASD systems.

Drug–polymer intermolecular interactions critically govern ASD performance [31,39]. Systems exhibiting homogeneous mixing and specific interactions (e.g., hydrogen bonding) demonstrate rapid drug dissolution proportional to polymer dissolution [40,41]. Conversely, systems lacking robust drug–polymer interactions show slow, polymer-independent dissolution [40]. Given their distinct physicochemical properties, surfactants modulate ASD dissolution through varied drug/polymer interaction mechanisms. Consequently, elucidating ternary drug–polymer–surfactant interactions provides a theoretical framework to unravel dissolution mechanisms and establish fundamental in vitro-in vivo correlations.

Hydrogen bonding and π-π stacking between PTX molecules stabilize its crystalline lattice, yielding low aqueous solubility [36]. Disrupting these noncovalent bonds represents a viable solubility enhancement strategy. Here, HPMC-AS disrupts drug–drug hydrogen bonding, establishing specific drug–polymer interactions instead. The incorporated surfactants modulated PTX/HPMC-AS ASD through distinct mechanisms. SLG and P188 were incompatible with PTX or HPMC-AS, forming no detectable interactions between their components. Tween 80 formed hydrogen bonding with PTX in both solution state and solid states; however, it didn’t disrupt the π-π stacking between PTX molecules. In contrast, NaTC, SLS, and Brij-35 were compatible with the PTX/HPMC-AS system. These surfactants facilitated the formation of ternary drug–polymer–surfactant interactions, and disrupted π-π stacking of PTX molecules. These differential ternary interactions directly governed the subsequent in vitro dissolution performance and in vivo bioavailability.

ASD dissolution involves simultaneous interdependent processes upon aqueous contact. Incorporated surfactants modulate each dissolution step [11,16], making deconstruction of these processes and monitoring of dynamic physicochemical changes essential for mechanistic elucidation. During dissolution, rapid wetting enables swift amorphous drug dissolution, generating supersaturation [40,42]. Optimal performance requires both high supersaturation and adequate maintenance duration. However, excessive supersaturation may accelerate precipitation rather than enhance absorption [27], while undissolved ASD matrices exhibit high crystallization propensity [38]. Therefore, systematic investigation of surfactant impacts must address: apparent solubility enhancement, supersaturation generation/stabilization, and crystallization inhibition of precipitates.

HPMC-AS has been recognized as a highly suitable polymer for maintaining PTX supersaturation and inhibiting crystallization in in vitro, and HPMC-AS-based PTX ASDs have been shown to significantly enhance the dissolution rate and, consequently, the oral bioavailability of the drug [12]. In line with this, we also selected HPMC-AS as the polymeric carrier to prepare a PTX/HPMC-AS ASD, which was subsequently physically blended with various surfactants to systematically evaluate their influence on dissolution behavior and oral bioavailability.

In this study, two types of PTX ASDs were prepared using distinct methods for specific applications. High drug-loading ASDs, with surfactant incorporated during preparation, were used for FT-IR and NMR studies to investigate drug–polymer–surfactant interactions. This approach maximized detection sensitivity for identifying fundamental molecular interactions. For dissolution and pharmacokinetic studies, lower drug-loading ASD formulations with surfactants added via physical blending were employed. This design reflects practical formulation strategies and enhances dissolution performance through a higher polymer content. Although the formulations differed, the results demonstrate clear correlations. Surfactants exhibiting weak molecular interactions (SLG, P188) had minimal effects on dissolution and bioavailability. Conversely, surfactants demonstrating strong compatibility (NaTC, Brij-35, SLS) significantly enhanced both dissolution performance and bioavailability, while Tween 80 induced crystallization despite an initial solubility enhancement, as discussed below.

Our findings indicate that surfactants physically blended with PTX/HPMC-AS ASD modulate interfacial processes such as wetting, thereby influencing dissolution performance. Upon immersion in the dissolution medium, the concurrent release of drug, polymer, and surfactant creates a dynamic solution environment where intermolecular interactions (e.g., hydrophobic association, micellar incorporation, or polymer-surfactant complexation) can occur. These interactions, in turn, influence key aspects of solution behavior such as supersaturation generation and maintenance, as well as the physical state of drug precipitates, collectively governing the in vivo bioavailability of the ASD.

HPMC-AS significantly enhanced crystalline PTX solubility by disrupting drug–drug hydrogen bonding and establishing drug–polymer interactions. It also helped maintain moderate supersaturation and inhibit precipitate crystallization. Surfactant effects were structure-dependent. SLG and P188 showed no detectable interactions, failed to improve solubility or modify dissolution. Tween 80 acted synergistically with HPMC-AS to enhance crystalline PTX solubility by disrupting hydrogen bonding. Similarly, SLG, NaTC, and Brij-35 also showed synergism with HPMC-AS, enhancing crystalline PTX solubility by disrupting hydrogen bonding and π-π stacking. However, their impact on dissolution performance diverged significantly: TW80 induced crystallization of the drug precipitates, and leading to incomplete and slow dissolution, inferior supersaturation generation, and ultimately the worst dissolution performance. In contrast, NaTC, SLS, and Brij-35 demonstrated optimal dissolution: rapid supersaturation generation, prolonged supersaturation maintenance, and extended amorphous stability of precipitates.

Pharmacokinetic studies revealed differential surfactant impacts: SLG and P188 exerted negligible effects, Tween 80 significantly reduced bioavailability (*p* < 0.001), while NaTC, SLS, and Brij-35 significantly enhanced it (*p* < 0.001). These findings confirm that surfactant effects on bioavailability directly correlate with their modulation of dissolution processes, including matrix wetting, supersaturation generation/maintenance, and precipitate crystallization inhibition. Critically, the ternary drug–polymer–surfactant interplay governs each dissolution stage, thereby determining ultimate bioavailability.

## 5. Conclusions

This study provides the first comprehensive analysis of surfactant impacts on in vitro dissolution, in vivo bioavailability, and their intercorrelations for PTX/HPMC-AS ASDs. We demonstrate that surfactants modulate ASD performance through multifaceted mechanisms. The overall performance is governed by a combination of surfactant properties, including their interfacial activity and their dynamic interactions with the drug and polymer in solution. Our key findings reveal three distinct functional categories of surfactants. Synergistic surfactants (e.g., NaTC, SLS, and Brij-35) enhance wetting and dissolution kinetics, generating high drug supersaturation while prolonging its maintenance and stabilizing amorphous precipitates, which collectively maximize bioavailability. Competitive surfactants (e.g., Tween 80) induced crystallization of the drug, thereby decreasing dissolution performance and reducing bioavailability. Neutral surfactants (e.g., SLG and P188) exhibit negligible interactions with drug or polymer, exerting minimal effects on dissolution or bioavailability. Rational ASD design thus requires thorough characterization of ternary interactions. We propose a multidimensional surfactant assessment framework focusing on: (a) Supersaturation generation and maintenance capacity. (b) Crystallization inhibition efficacy in precipitates. This mechanistic understanding establishes a predictive basis for surfactant selection to optimize ASD performance.

## Figures and Tables

**Table 1:** Solubility of crystalline PTX in PBS with HPMC-AS, surfactant, and their combinations (mean ± SD, *n* = 3). Color scale: green (minimum), yellow (intermediate), red (maximum solubility).

Paclitaxel Solubility (μg/mL)								
Medium	Without Surfactants	With Surfactants	SLG	NaTC	SLS	TW80	P188	Brij-35
PBS	< 0.1	1mg/mL	1.2 ± 0.3	3.6 ± 1.2	3.5 ± 0.8	2.5 ± 0.7	1.1 ± 0.3	1.7 ± 0.2
		2mg/mL	1.1 ± 0.2	10.1 ± 3.4	17 ± 1.9	4.5 ± 0.5	2.2 ± 0.3	3.5 ± 0.5
		4mg/mL	1.3 ± 0.2	9.3 ± 1.7	15.4 ± 1.9	9 ± 1.4	2.4 ± 0.2	6.7 ± 1.3
2mg/mL HPMC-AS	6.6 ± 0.5	1mg/mL	7.9 ± 1.5	13.8 ± 1.0	20.9 ± 1.4	18.8 ± 1.4	6.9 ± 0.4	21.1 ± 3.1
4mg/mL HPMC-AS	8.6 ± 1.8	2mg/mL	11.2 ± 1.9	20.1 ± 3.6	73.9 ± 5.7	45.7 ± 8.6	8.2 ± 2.2	85.2 ± 8.2
8mg/mL HPMC-AS	10.7 ± 1.6	4mg/mL	12.0 ± 2.5	20.3 ± 2.0	177.3 ± 2.6	132.4 ± 27.1	10.6 ± 1.2	128.3 ± 19.5
Solubility(μg/mL)	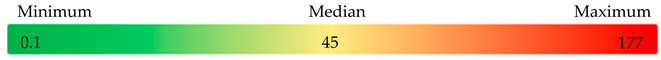							

**Table 2:** Pharmacokinetic parameters derived from non-compartmental analysis of PTX after oral administration of different formulations (40 mg/kg eq.) in rats.

Formulation	Parameters				
	C_max_/(ng/mL)	T_max_/h	T_1/2_/h	AUC_0-t_(μg·h/L)	MRT_0-t_/h
Crystalline PTX	20.6 ± 2.7	0.8 ± 0.0	3.7 ± 3.5	118.5 ± 22.7	5.9 ± 1.4
ASD	140.6 ± 19.9 ^###^	1.0 ± 0.0	7.7 ± 3.8	1231.2 ± 118.3 ^###^	7.2 ± 0.6
ASD+SLG	128.3 ± 7.6 ^ns^	0.9 ± 0.1	4.6 ± 3.5	1281.3 ± 230.9 ^ns^	8.0 ± 0.7
ASD+NaTC	351.9 ± 35.7 ***	1.0 ± 0.0	6.0 ± 4.5	2849 ± 422.2 ***	7.2 ± 0.8
ASD+SLS	459.6 ± 43.7 ***	0.8 ± 0.0	3.3 ± 1.9	2899.8 ± 148.3 ***	5.2 ± 0.5
ASD+TW80	59.7 ± 2.2 ***	1.0 ± 0.0	17.1 ± 4.1	865.8 ± 42.8 ***	8.8 ± 0.8
ASD+P188	124.0 ± 12.8 ^ns^	1.0 ± 0.0	11.5 ± 2.3	1420.4 ± 130.1 ^ns^	7.5 ± 1.2
ASD+Brij-35	361.1 ± 21.6 ***	0.8 ± 0.0	7.0 ± 1.9	2612.6 ± 261.8 ***	5.8 ± 0.4

**Abbreviations:** C_max_: maximum plasma concentration; T_max_: time to reach C_max_; T_1/2_: half-life; AUC: area under curve; MRT: mean residence time. ###: *p* < 0.001(crystalline PTX vs. PTX/HPMC-AS ASD, Student’s *t*-test); ns, not significant; ***: *p* < 0.001 (surfactant-free ASD vs. ASD + surfactant formulations, Student’s *t*-test) (mean ± SD, *n* = 5).

## Data Availability

The original contributions presented in this study are included in the article/Supplementary Materials. Further inquiries can be directed to the corresponding authors.

## Supplementary Materials

The following supporting information can be downloaded at: https://www.mdpi.com/article/10.3390/pharmaceutics17111487/s1, Table S1: Plasma concentrations of PTX over time in individual animals after oral administration of different formulations (*n* = 5).
