# Supplementary material for: Impact of Different Surfactants on Oral Bioavailability of Paclitaxel/HPMC-AS Amorphous Solid Dispersion
Source: Pharmaceutics. 2025 Nov 18;17(11):1487. doi: 10.3390/pharmaceutics17111487 (PMC12655036; doi:10.3390/pharmaceutics17111487)
Supplement: Supplementary file 1 [file pharmaceutics-17-01487-s001.zip › pharmaceutics-3859485-supplementary.pdf]

Article

Impact of Different Surfactants on Oral Bioavailability of Paclitaxel/HPMC-AS Amorphous Solid Dispersion

Chenzhao Zhang [1,†], Siyi Mao [1,†], Jinhua Yuan [1], Xiuzhen Ma [1], Aiya Xing [1], Xiaoling Liu [2,*] and Yuejie Chen [1,3,*]

[1] School of Pharmacy, Minzu University of China, Beijing 100081, China; 23302008@muc.edu.cn (C.Z.); 24302083@muc.edu.cn (S.M.); 23302010@muc.edu.cn (J.Y.); 25302185@muc.edu.cn (X.M.); 22301902@muc.edu.cn (A.X.)
[2] School of Life Sciences, Beijing University of Chinese Medicine, Beijing 102401, China
[3] Key Laboratory of Mass Spectrometry Imaging and Metabolomics (Minzu University of China), State Ethnic Affairs Commission, Beijing 100081, China
* Correspondence: liuxiaoling66@bucm.edu.cn (X.L.); chenyuejie@muc.edu.cn (Y.C.); Tel.: +86-(010)-6893-9942
† Chenzhao Zhang and Siyi Mao are co-first authors of this article.

Supplementary Materials

Table S1. Plasma concentrations of PTX over time in individual animals after oral administration of different formulations (*n* = 5).

Plasma concentrations of PTX over time in individual animals after oral administration of different formulations (n=5)						
**PTX formulation**	**Con.(ng/mL)**					
	**Time(h)**	**Rat1**	**Rat2**	**Rat3**	**Rat4**	**Rat5**
**(F_1)Cystalline PTX**	0.17	7.4	7.4	4.9	4.9	4.7
	0.33	13.6	13.6	10.1	14.1	13.2
	0.50	15.8	15.8	14.5	22.3	14.6
	0.75	19.5	19.5	17.6	23.9	20.7
	1	18.2	18.2	14.1	18.6	18.9
	1.5	15.0	15.0	12.2	12.2	13.3
	2	10.6	10.6	10.3	9.6	9.8
	4	5.4	5.4	5.2	8.7	8.4
	24	1.3	1.3	0.7	1.8	1.4
	**Time(h)**	**Rat1**	**Rat2**	**Rat3**	**Rat4**	**Rat5**
**(F_2)ASD**	0.17	21.8	25.7	17.3	21.0	18.6
	0.33	58.5	49.0	51.5	63.2	50.3
	0.50	104.0	76.5	96.6	123.7	88.9
	0.75	143.9	110.7	118.4	150.7	117.7
	1	150.2	120.9	127.4	163.8	133.5

	Time(h)	Rat1	Rat2	Rat3	Rat4	Rat5
	1.5	123.7	112.9	118.4	154.8	117.4
	2	103.4	90.3	80.7	118.3	123.8
	4	53.9	68.6	61.3	67.9	70.3
	24	19.0	20.8	27.6	27.8	20.1
**(F_3)ASD+SLG**	**Time(h)**	**Rat1**	**Rat2**	**Rat3**	**Rat4**	**Rat5**
	0.17	23.9	33.2	23.5	30.1	21.9
	0.33	69.9	80.3	69.9	80.0	70.4
	0.50	101.5	117.7	105.2	114.4	102.2
	0.75	117.5	133.4	124.6	123.5	118.3
	1	119.6	127.9	132.1	126.6	118.5
	1.5	97.2	113.7	117.9	113.2	99.3
	2	73.9	104.7	90.8	101.1	77.7
	4	47.0	83.3	64.8	81.1	44.3
	24	28.3	28.8	28.5	26.6	25.1
**(F_4)ASD+NaTC**	**Time(h)**	**Rat1**	**Rat2**	**Rat3**	**Rat4**	**Rat5**
	0.17	40.9	47.8	55.2	74.0	43.3
	0.33	141.3	133.4	129.1	194.3	155.7
	0.50	241.0	229.6	244.8	237.6	239.9
	0.75	280.2	271.3	277.2	344.4	280.6
	1	350.4	302.1	380.4	374.8	377.5
	1.5	333.9	233.5	326.6	328.0	334.2
	2	209.1	189.8	271.6	194.2	197.6
	4	133.5	126.9	188.1	133.8	127.4
	24	36.8	58.1	60.4	62.0	34.8
**(F_5)ASD+SLS**	**Time(h)**	**Rat1**	**Rat2**	**Rat3**	**Rat4**	**Rat5**
	0.17	174.3	153.9	178.9	172.5	171.1
	0.33	357.5	322.2	262.0	272.5	356.4
	0.50	448.5	404.9	415.7	394.8	442.3
	0.75	500.0	452.1	485.0	401.3	467.7
	1	461.1	395.0	378.2	382.1	360.8
	1.5	317.8	322.2	327.1	324.9	332.5
	2	283.0	262.9	260.2	268.8	277.2
	4	156.1	136.7	157.8	167.7	135.9
	24	24.6	28.7	41.1	25.4	27.3
**(F_6)ASD+TW80**	**Time(h)**	**Rat1**	**Rat2**	**Rat3**	**Rat4**	**Rat5**
	0.17	35.3	25.8	27.2	22.7	23.7
	0.33	49.2	43.2	49.6	44.6	47.0
	0.50	50.8	53.1	54.1	48.1	53.2
	0.75	55.3	58.6	57.9	55.7	54.2
	1	54.4	62.3	63.0	59.2	58.5
	1.5	51.9	61.0	61.7	57.1	57.4
	2	47.4	55.9	59.1	52.6	54.0
	4	39.1	44.7	45.3	43.2	46.9
	24	16.5	23.2	19.2	26.7	28.7
**(F_7)ASD+P188**	**Time(h)**	**Rat1**	**Rat2**	**Rat3**	**Rat4**	**Rat5**
	0.17	26.7	18.8	33.5	25.4	23.3
	0.33	81.3	71.5	71.3	72.3	70.9

	0.50	109.9	102.1	83.2	88.3	108.6
	0.75	133.6	123.7	97.9	99.8	96.6
	1	140.7	126.9	116.6	111.7	113.9
	1.5	122.5	105.9	113.6	107.6	112.4
	2	114.6	93.5	92.0	86.8	89.6
	4	99.4	70.6	65.9	74.3	76.4
	24	17.8	29.5	30.9	33.1	16.9
	**Time(h)**	**Rat1**	**Rat2**	**Rat3**	**Rat4**	**Rat5**
	0.17	40.8	59.4	64.9	40.5	49.2
	0.33	117.0	115.7	145.2	133.1	123.4
	0.50	332.0	274.8	282.2	252.1	261.4
**(F_8)ASD+Brij-35**	0.75	405.0	352.7	375.0	322.3	350.5
	1	390.4	307.6	313.1	267.6	290.1
	1.5	268.2	281.4	252.9	249.2	271.8
	2	214.4	242.6	208.7	199.9	212.2
	4	120.6	180.3	161.7	130.8	136.2
	24	36.9	29.0	28.5	34.8	32.9
